# Effect of the Location of Dental Mini-Implants on Strain Distribution under Mandibular Kennedy Class I Implant-Retained Removable Partial Dentures

**DOI:** 10.1155/2021/6688521

**Published:** 2021-05-04

**Authors:** Pimduen Rungsiyakull, Kallaya Kujarearntaworn, Pathawee Khongkhunthian, Michael Swain, Chaiy Rungsiyakull

**Affiliations:** ^1^Department of Prosthodontics, Faculty of Dentistry, Chiang Mai University, Chiang Mai, Thailand; ^2^Muang Sam Sip Hospital, Ubon Ratchathani, Thailand; ^3^Center of Excellence in Dental Implantology, Faculty of Dentistry, Chiang Mai University, Chiang Mai, Thailand; ^4^Biomaterials Research Unit, Faculty of Dentistry, The University of Sydney, Sydney, Australia; ^5^Department of Mechanical Engineering, Faculty of Engineering, Chiang Mai University, Chiang Mai, Thailand

## Abstract

**Purpose:**

To investigate the effect of minidental implant location on strain distributions transmitted to tooth abutments and dental minidental implants under mandibular distal extension removable partial denture.

**Materials and Methods:**

A mandibular Kennedy Class I distal extension model missing teeth 35–37 and 45–47 was constructed. Six dental mini-implants were placed at positions A, B, and C, where position A was 6.5 mm distal to the abutment teeth with 5 mm between each position. Fourteen uniaxial strain gauges were bonded on the model at the region of dental mini-implant and abutment (first premolar). Four groups were designated according to the location of the mini-implants. A load of 150 N and 200 N was applied using an Instron testing machine. Loadings consisted of bilateral and unilateral loading. Comparisons of the mean microstrains among all strain gauges in all situations were analyzed.

**Results:**

Variation in mini-implant locations induced local strains in different areas. Strains at the tooth abutment were significantly decreased in the group in which implants were placed mesially. Strains around the mini-implants showed different patterns when loaded with different loading conditions. The group in which implants were placed distally showed the lowest strains compared to other groups.

**Conclusion:**

Mesially placed mini-implants showed the lowest strain around abutment teeth, while a distally-placed mini-implants presented the lowest strain around mini-implants themselves. Under favorable biting force, mini-implant is an option to assist mandibular distal extension removable partial denture. Mesially placed mini-implants are recommended when the abutment has periodontally compromised conditions and a distally placed mini-implant when periodontal conditions are stable.

## 1. Introduction

To replace missing teeth with removable partial dentures (RPDs) is an option, especially when the area of missing teeth is a long-span arch or distal free-end extension. However, teeth and soft tissue differ in resilience. The soft tissue under load has a displacement range of 350–500 *µ*m, whereas teeth have 20 *µ*m at the same loading [1]. For this reason, when force is applied to prostheses in the posterior area, the prostheses are usually displaced toward the soft tissue. As a result of prostheses being displaced toward the soft tissue, the residual ridge sustains trauma due to heavy torsional stress from denture movement. The combination of removable partial dentures and implants may reduce vertical displacement of the prosthesis, providing significantly greater amounts of force and decreased stress around abutment teeth when compared with conventional removable partial dentures [2, 3].

Many factors, such as surface treatment, type, number, length, diameter, and location of implants, influence force transfer and subsequent stress distribution around the marginal bone level of implants and implant components [4, 5]. As regards the location of implants, several studies have examined RPDs combined with standard implants using finite element analysis (FEA) and strain gauge analysis. Those studies revealed that RPDs assisted by implants located mesially in the edentulous space demonstrated the highest stresses around the implants and the abutment teeth [6–8].

In some edentulous areas that have limitations, such as inadequate bone volume, length, and height of the ridge, dental mini-implants are recommended instead of standard implants. Dental mini-implants are easy to place, have low cost, and use minimally invasive surgical procedures [9]. However, the implant diameter has an influence on stress distribution along the bone-implant interface, as investigated by Misch and Bidez [10]. Implant diameter has a greater effect on stress distribution in bone than does implant length or geometry [11]. Many reports indicate that an increase in implant diameter decreases the maximum stress around the implant neck [11–14]. Wider-diameter implants have increased surface area. Thus, wider implants can distribute force through greater surface areas [10]. In fixed partial dentures, dental mini-implants have greater strain values than standard supporting implants [15]. In complete denture, the use of a low number of minidental implants tends to produce low strain values in the retromolar denture-bearing area and around the terminal MDIs during posterior loadings [16]. Nevertheless, there is no study to date on dental mini-implants combined with removable partial dentures. Therefore, the purpose of this study was to analyze strain distribution on the first premolar abutments, on the simulated bone to the mesial and distal sides of dental mini-implants, and on the end-surface of the model under implant-retained mandibular Kennedy Class I removable partial dentures in various locations.

## 2. Materials and Methods

A mandibular Kennedy Class I bilateral distal extension model missing teeth 35–37 and 45–47 was constructed of two components: self-polymerizing acrylic (Vertex Self Curing, Soesterberg, Netherlands) and silicone (GI Mask, Coltene, Madrid, Spain). The self-polymerizing acrylic simulated hard tissue. The model was covered with a silicone layer that simulated human soft tissue. The ranges of Shore hardness A of silicone material (14 to 16) are similar to that of human soft tissue (16 to 21) [17]. The thickness of the silicone material was 2 mm. The 34–44 artificial teeth (PE-ANA002®; Nissin, Kyoto, Japan) had an artificial periodontal ligament, which was simulated by silicone impression material (GI Mask). The thickness of PDL substitutes was between 0.25 and 0.3 mm [18]. The conventional cobalt-chrome-molybdenum Kennedy Class I RPD was fabricated to the model. The RPD was composed of a lingual bar and rest-proximal plate–Akers clasps on both first premolar abutments. The free-end saddles were fabricated to the occlusal rim using acrylic resin.

Six dental mini-implants (size 2.75 × 10 mm, PW+, Nakhon Pathom, Thailand) were placed at positions A, B, and C, where position A was 6.5 mm distal to the abutment with 5 mm between each position and the other.

**Figure 1:**
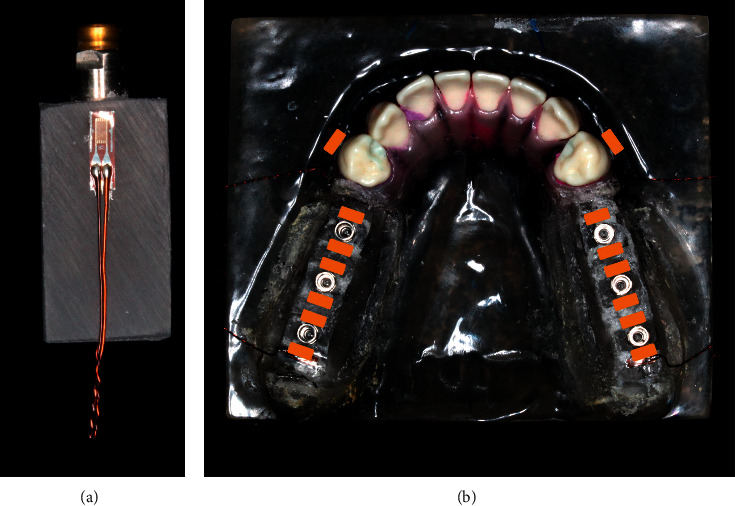
(a) Dental mini-implant attached to a strain gauge. (b) Model labeled with the locations of strain gauges.

Fourteen uniaxial strain gauges were used to make the measurements in this study (Figure 1). Twelve strain gauges (KFG-1N-120-C1-11N50C2; Kyowa Electronic Instruments Co, Ltd, Tokyo, Japan) were bonded on the mesial and distal surfaces of the model adjacent to the first thread region of each dental mini-implant, 1 mm away from the implant body and perpendicular to the occlusal plane with a thin film of methyl-2-cyanoacrylate resin. Two strain gauges were bonded on the buccal surface and parallel to the long axis of the primary abutment (first premolar).

**Figure 2:**
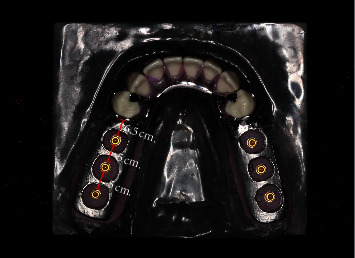
Model of mandibular Kennedy Class I bilateral distal extension missing (35–37 and 45–47) with a framework.

Four groups were designated according to the location of the dental mini-implants (Figure 2):

  Group 1: a conventional distal extension RPD, replacing 35–37 and 45–47.
  Group 2: a conventional distal extension RPD with dental mini-implant at position A
  Group 3: a conventional distal extension RPD with dental mini-implant at position B.
  Group 4: a conventional distal extension RPD with dental mini-implant at position C.

Retentive caps with a diameter of 4.4 mm and that required a removal force of 0.6 kg (Ø 4.4 × 0.6 kg) were selected. When testing Group 1, all abutments (equator) of dental mini-implants were removed. In Groups 2, 3, and 4, as in Group 1, the abutments of dental mini-implants that were not tested were removed. Equator attachments were placed on the implants that were tested.

A load of 150 N and 200 N were applied at a crosshead speed of 0.05 mm/sec for 15 seconds using an Instron universal testing machine (Instron, Norwood, MA, USA) [19]. Loadings consisted of bilateral loading and unilateral loading. A bilateral loading was applied on four points: at the center point between positions A and B and B and C on each side. Four stainless steel balls (4.0 mm in diameter) were located on these points on the acrylic occlusal rim and soldered to a wide brass plate (approximately 6 cm × 3 cm *x* 0.5 cm). For central loading, another stainless steel ball (12.5 mm in diameter) was attached at the center of the plate [20].

**Figure 3:**
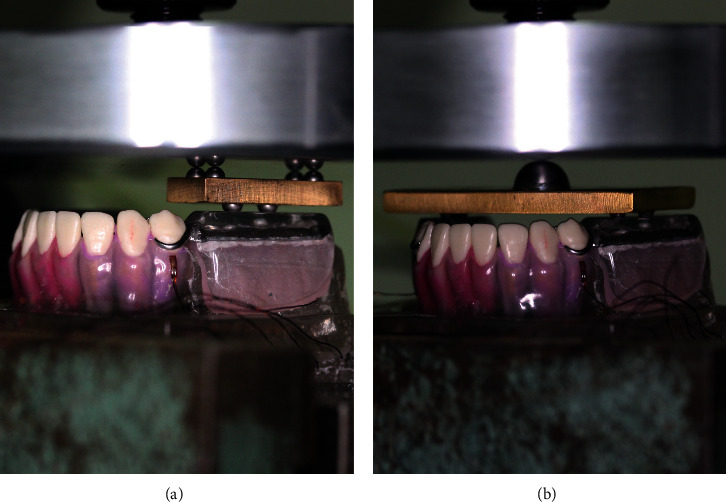
Axial loading. (a) Bilateral loading using a wide brass plate and stainless steel balls. (b) Unilateral loading using a narrow brass plate and stainless steel balls.

For unilateral loading (performed on each side), two stainless steel balls were positioned at the center points between positions A and B and B and C on one side and were soldered to a narrow brass plate (approximately 1.5 cm × 3 cm *x* 0.5 cm). Another four stainless steel balls were placed on the opposite side (Figure 3). To offer sufficient statistical power, each loading condition was repeated 9 times [8]. Before each loading, all of the strain gauges were set at zero.

Data were collected for microstrain values by each strain gauge and presented as mean and standard deviation (SD) values. The significance level was set at *P* ≤ 0.05. Statistical analysis was performed using SPSS version 17.0 (SPSS, Inc., Chicago, IL, USA). One-way ANOVA was used to analyze mean strain values, and the multiple comparisons test was used to compare the results.

## 3. Results

**Figure 4:**
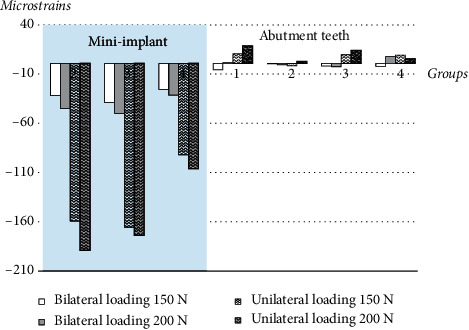
Comparison of microstrains around abutment teeth and dental mini-implants in all loading conditions with 150 N and 200 N bilateral and loading.

Microstrain values were presented in two different areas: abutment teeth and dental mini-implants. The strain values of the dental mini-implants were higher than those of the abutment teeth in all conditions (Figure 4). The differences in microstrain values were significantly greater at the dental mini-implant areas, whereas there were no significant differences in microstrain values at the abutment teeth. The higher load applied to the model, the greater differences in microstrains were found.

Microstrain values in the four groups under applied bilateral loading of 150 N and 200 N are presented in Table 1. No statistically significant differences were found in microstrains at the abutment teeth among all groups. However, there were significant differences in microstrains at the dental mini-implants. Group 4 presented the lowest microstrains.

Microstrains were recorded at the loaded and unloaded sites when unilateral loads of 150 and 200 N were applied (Table 2). At the loaded site, Group 2 showed the lowest compressive microstrains on the abutment teeth, whereas other groups presented higher tensile microstrains. Meanwhile, Group 4 showed statistically significant differences in microstrains, with the lowest values at the dental mini-implants. At the unloaded site, there was no statistically significant difference in microstrains at the abutment teeth, whereas at the implants, significant differences in tensile strains were found only in Group 2.

**Figure 5:**
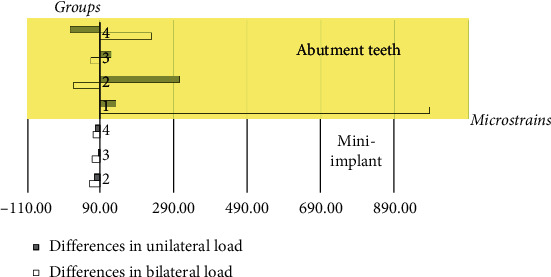
Differences in microstrain values when the load was increased from 150 N to 200 N.

When the applied load increased from 150 N to 200 N, there were differences in strain values among two different locations: the abutment teeth and the dental mini-implants. Strain values were affected by the difference in location of the dental mini-implants. Distally placed dental mini-implants induced less microstrain at the implants than did those placed mesially. In addition, higher loading force tended to increase compressive microstrains at the dental mini-implant up to approximately 5–40%, whereas no trends in strain values were found at the abutment teeth (Figure 5).

## 4. Discussion

Strains on bone, abutment teeth, and implants are some of the factors that indicate the long-term success of implant-retained RPDs. Strains over the threshold values cause de-osseointegration of implants, peri-implant bone loss, abutment tooth mobility, and fracture of prostheses [21–24]. Implant placements may reduce soft tissue intrusion of the distal-extension RPD, and the location of the placement has an influence on the number of strains [8]. In this study, it was observed that distally placed implants resulted in greater strain transmission along the implants themselves, whereas mesially placed implants presented less strain transmission at the abutment teeth. The cause of this difference in strain transmission may be the elimination of the cantilever as a result of the distal placement of the implant. Therefore, a tooth-tissue supported prosthesis is converted into a bounded-tooth, implant-supported prosthesis [6, 25, 26].

**Figure 6:**
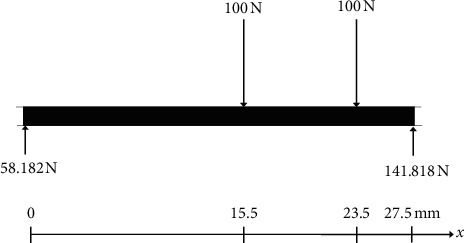
Free-body diagram demonstrating base length as represented by the sum of each replacement tooth and implant.

Many loading situations can simplify strain analysis. With different implant-placement locations, the abutment teeth and implants were subjected to loads, which resulted in different patterns of strains. To verify the experimental results, a theoretical two-dimensional equilibrium analysis was conducted through a diagram of an isolated mechanical system considered as a single body which is known as a free-body diagram. The free-body diagram was modelled as a simple beam that represents unilateral mandibular implant-retained RPDs. Thus, the supporting force at both ends of the beam was modelled as pin support on the tooth support side and as roller support on the implant supported side. However, please noted that the horizontal force components are not considered since the applied forces have only a vertical component (Figure 6) [27]. The forces applied at the beam represented all of the forces applied to the system, which are the sum of the mesiodistal dimensions of each replacement tooth (first premolar: P1) and of the implants placed at position A, B, or C (M1), and loading force at two positions between P1 and M1 (100 N vertical load were set at 15.5 mm and 23.5 mm next to the P1 location, respectively). Shear forces and bending moments were then calculated using the free-body diagram and equations of equilibrium equations (1) and (2), as follows [28]:

(1) $$\begin{array}{c} \sum^{}F=0,\;P-V\;=\;0, \end{array}$$

(2) $$\begin{array}{c} \sum^{}M=0,\;M-Px=0, \end{array}$$

*F* = force (*N*), *P* = load (*N*), V = shear force (*N*), *M* = bending moment Nmm, and *x* = distance (mm) from the end of the beam to the cross section where *V* and *M* are being determined.

The shear forces and peak bending moments for all three different implant positions are described in Table 3. Implant position, loading location, and pattern of loading indicated the shear force and bending moment values around the abutment tooth, implant, and dental prosthesis. The shear forces represented the shear stress acting in a transverse direction to the occlusal plane [28]. From this theoretical model, it was shown that the lowest shear force at the implant was present when placing the implant at position C. This theoretical finding was similar to the experimental results, in which strains at the dental mini-implants in Group 4 were the lowest. There was also less tensile strain at the unloaded site than at the loaded site under unilateral loading in Group 4. The explanation for this finding is that placing the implant at position C eliminated the cantilever length and converted the denture from being a tooth-tissue prosthesis to a tooth-implant-retained prosthesis, reducing the shear force at the implant itself [6]. As regards the abutment tooth, the shear forces were lowest when the implant was placed at position B in the theoretical study instead of at position A as presented in the experimental test. This finding may relate to the short distance between the implant position and the location of the applied occlusal force. Mesially placed implants close to the abutment tooth may change the fulcrum point from the rest seat at the abutment to the implant. This mesial placement resulted in the reduction of vertical displacement of the denture and, consequently, less stress being transferred to the abutment tooth [29].

In terms of the peak bending moments, the theoretical result revealed that the magnitude of the moment conformed to the shear forces at the abutment tooth and at the implant. Bending moment is a resultant of shear force as a function of distance and negatively affects the stability of the distal extension RPD [28]. The higher the bending moment developed, the more the prosthesis can be displaced against the supporting elements of the denture [30]. The peak bending moment showed the lowest value when an implant was placed at position B. The stability of the denture may be related to the pattern of loading [31]. In this study, bilateral loading showed lower strain values than did unilateral loading. Bilateral loading created less lateral and vertical displacement of the denture, generating less tensile strain at the interface between the metal and acrylic surfaces of the denture [31, 32]. Placing a dental mini-implant at position A provided more favorable strain distribution around the abutment tooth, whereas placing a dental mini-implant at position C transferred less strain at the implant itself. The authors recommend placing the minidental implant at position A when the abutment has periodontally compromised conditions and at position C when periodontal conditions are stable. These recommendations are in agreement with the findings of Gonçalves et al. [23], who reported overall satisfactory results with the use of distal implant-retained RPD overdentures.

The implant diameter has an influence on stress distribution along the bone-implant interface [4, 10, 33]. A decrease in implant diameter may increase the stress transferred to the bone-implant contact [15]. In the range of 3.3 to 5.3 mm diameter implants, for every 0.5 mm decrease in width, there is a 10%–15% decrease in surface area. This percentage change is greater for smaller diameters and less for larger diameters [15]. Although strain values reported in this study did not exceed the physiologic loading zone, which has been reported in the range of 1,000–3,000 microstrains [21], the actual transfer of magnitude data needed to have been considered because this was an *in vitro* study. In an *in vivo* study, material (bone, tooth, etc.) properties would be different. The use of mini-implants may lead to a higher implant failure rate than if standard implants are used, especially in cases with heavy bite forces, such as patients with parafunctional habits, long-span edentulous ridges, and cases with opposing natural dentitions [34]. Therefore, to minimize the bending overload beyond the threshold yield strength of dental mini-implant materials, increasing the number of dental mini-implants could be an option. Further study is needed to determine the effects of the number and location of dental mini-implants in relation to the situation that has greater biting forces under mandibular Kennedy Class I implant-retained RPDs.

## 5. Conclusions

1. The location of dental mini-implant placement has an influence on strain distribution in abutment teeth and the dental mini-implant itself.
2. A mesially placed dental mini-implant decreased microstrains around abutment teeth compared to a distally placed dental mini-implant.
3. A distally placed dental mini-implant presented decreased microstrains around the dental mini-implant itself.
4. Microstrain values for dental mini-implants under applied bilateral load were lower than under unilateral load.

## Figures and Tables

**Table 1:** Mean microstrain of abutment teeth and dental mini-implants under applied bilateral loading (mean strain values (±SD in microstrain)).

Group	Implants		Abutment teeth	
	150 N	200 N	150 N	200 N
1	—	—	−6.92 (10.11)^a^	3.87 (10.48)^a^
2	−34.79 (8.77) ^ab^	−48.69 (8.22)^a^	−0.45 (5.24)^a^	−1.52 (9.66)^a^
3	−42.50 (13.35)^a^	−54.11 (13.19)^a^	−2.77 (11.60)^a^	−3.62 (17.30)^a^
4	−28.02 (11.05)^b^	−34.45 (5.77)^b^	−3.16 (14.29)^a^	7.65 (7.21)^a^

Comparisons done appear in the same column. The same upper superscript case letter in the same column indicates no significant difference at *P* = 0.05.

**Table 2:** Mean microstrain of abutment teeth and dental mini-implants under applied unilateral loading (mean strain values (±SD in microstrain)).

Group	Loaded site				Unloaded site			
	Implant		Abutment tooth		Implant		Abutment tooth	
	150 N	200 N	150 N	200 N	150 N	200 N	150 N	200 N
1	—	—	10.50 (6.83)^b^	19.04 (9.27)^c^	—	—	10.07 (9.89)^a^	7.23 (12.30)^a^
2	−170.65 (6.03)^a^	−202.32 (4.29)^a^	−2.48 (10.22)^a^	2.11 (12.12)^a^	14.48 (7.85)^b^	22.57 (5.09)^b^	0.21 (12.62)^a^	6.01 (7.56)^a^
3	−177.59 (11.61)^a^	−186.22 (12.37)^b^	9.78 (9.02)^ab^	14.29 (8.42)^bc^	−6.39 (14.81)^a^	4.07 (8.58)^a^	1.97 (15.93)^a^	2.11 (10.63)^a^
4	−99.12 (3.70)^b^	−113.84 (6.38)^c^	9.09 (4.29)^ab^	5.04 (6.45)^ab^	−10.85 (6.64)^a^	−0.06 (5.83)^a^	9.58 (10.95)^a^	2.82 (7.20)^a^

Comparisons done appear in the same column. The same upper superscript case letter in the same column indicates no significant difference at *P* = 0.05.

**Table 3:** Shear forces and peak bending moments developed in the free-body diagram.

Position of implant	*A*	*B*	*C*
Force at abutment (N)	−139.13	0	58.18
Force at implant (N)	339.13	200	141.82
Peak bending moment (Nmm)	−1600	−400	901.82

## Data Availability

The data used to support the findings of this study are available from the corresponding author upon request.

## References

[1] Manderson R. D., Wills D. J., Picton D. C. Biomechanics of denture-supporting tissues.

[2] Rodrigues R. C. S., Faria A. C. L., Macedo A. P., de Mattos M. d. G. C., Ribeiro R. F. (2013). Retention and stress distribution in distal extension removable partial dentures with and without implant association. *Journal of Prosthodontic Research*.

[3] Ohkubo C., Kobayashi M., Suzuki Y., Hosoi T. (2008). Effect of implant support on distal-extension removable partial dentures: in vivo assessment. *The International Journal of Oral & Maxillofacial Implants*.

[4] Sahin S., Cehreli M. C., Yalcin E. (2002). The influence of functional forces on the biomechanics of implant-supported prostheses-a review. *Journal of Dentistry*.

[5] Guarnieri R., Di Nardo D., Gaimari G., Miccoli L., Testarelli L. (2019). Short vs. standard laser-microgrooved implants supporting single and splinted crowns: a prospective study with 3 years follow-up. *Journal of Prosthodontics*.

[6] Xiao W., Li Z., Shen S., Chen S., Wang Y., Wang J. (2014). Theoretical role of adjunctive implant positional support in stress distribution of distal-extension mandibular removable partial dentures. *The International Journal of Prosthodontics*.

[7] Memari Y., Geramy A., Fayaz A., Rezvani Habib Abadi S., Mansouri Y. (2014). Influence of implant position on stress distribution in implant-assisted distal extension removable partial dentures: a 3D finite element analysis. *Journal of Dentistry (Tehran, Iran)*.

[8] Hegazy S. A., Elshahawi I. M., Elmotayam H. (2013). Stresses induced by mesially and distally placed implants to retain a mandibular distal-extension removable partial overdenture: a comparative study. *The International Journal of Oral & Maxillofacial Implants*.

[9] Christensen G. J. (2006). The “mini”-implant has arrived. *The Journal of the American Dental Association*.

[10] Misch C. E., Bidez M. W. (1999). *Contemporary Implant Dentistry*.

[11] Anitua E., Tapia R., Luzuriaga F., Orive G. (2010). Influence of implant length, diameter, and geometry on stress distribution: a finite element analysis. *The International Journal of Periodontics & Restorative Dentistry*.

[12] Himmlová L., Dostálová T. J., Kácovský A., Konvic̆ková S. (2004). Influence of implant length and diameter on stress distribution: a finite element analysis. *The Journal of Prosthetic Dentistry*.

[13] Tuncelli B., Poyrazoglu E., Köylüoglu A. M., Tezcan S. (1997). Comparison of load transfer by implant abutments of various diameters. *The European Journal of Prosthodontics and Restorative Dentistry*.

[14] Coelho Goiato M., Pesqueira A. A., Santos D. M., Haddad M. F., Moreno A. (2014). Photoelastic stress analysis in prosthetic implants of different diameters: mini, narrow, standard or wide. *Journal of Clinical and Diagnostic Research*.

[15] Sallam H., Kheiralla L. S., Aldawakly A. (2012). Microstrains around standard and mini implants supporting different bridge designs. *Journal of Oral Implantology*.

[16] Warin P., Rungsiyakull P., Rungsiyakull C., Khongkhunthian P. (2018). Effects of different numbers of mini-dental implants on alveolar ridge strain distribution under mandibular implant-retained overdentures. *Journal of Prosthodontic Research*.

[17] Thomas V. J., Patil K. M., Radhakrishnan S., Narayanamurthy V. B., Parivalavan R. (2003). The role of skin hardness, thickness, and sensory loss on standing foot power in the development of plantar ulcers in patients with diabetes mellitus-A preliminary study. *The International Journal of Lower Extremity Wounds*.

[18] Mühlemann H. R. (1967). Tooth mobility: a review of clinical aspects and research findings. *Journal of Periodontology*.

[19] Miyaura K., Morita M., Matsuka Y., Yamashita A., Watanabe T. (2000). Rehabilitation of biting abilities in patients with different types of dental prostheses. *Journal of Oral Rehabilitation*.

[20] Hirajima Y., Takahashi H., Minakuchi S. (2009). Influence of a denture strengthener on the deformation of a maxillary complete denture. *Dental Materials Journal*.

[21] Isidor F. (2006). Influence of forces on peri-implant bone. *Clinical Oral Implants Research*.

[22] Mijiritsky E., Lorean A., Mazor Z., Levin L. (2015). Implant tooth-supported removable partial denture with at least 15-year long-term follow-up. *Clinical Implant Dentistry and Related Research*.

[23] Gonçalves T. M. S. V., Campos C. H., Rodrigues Garcia R. C. M. (2014). Implant retention and support for distal extension partial removable dental prostheses: satisfaction outcomes. *The Journal of Prosthetic Dentistry*.

[24] Kaufmann R., Friedli M., Hug S., Mericske-Stern R. (2009). Removable dentures with implant support in strategic positions followed for up to 8 years. *The International Journal of Prosthodontics*.

[25] Ohkubo C., Kurihara D., Shimpo H., Suzuki Y., Kokubo Y., Hosoi T. (2007). Effect of implant support on distal extension removable partial dentures: in vitro assessment. *Journal of Oral Rehabilitation*.

[26] Mijiritsky E. (2007). Implants in conjunction with removable partial dentures: a literature review. *Implant Dentistry*.

[27] Oh W. S., Oh T. J., Park J. M. (2016). Impact of implant support on mandibular free-end base removable partial denture: theoretical study. *Clinical Oral Implants Research*.

[28] James M. (1997). *Gere SPT. Mechanics of Materias*.

[29] Cunha L. D. A. P., Pellizzer E. P., Verri F. R., Pereira J. A. (2008). Evaluation of the influence of location of osseointegrated implants associated with mandibular removable partial dentures. *Implant Dentistry*.

[30] Monteith B. D. (1984). Management of loading forces on mandibular distal-extension prostheses. Part I: evaluation of concepts for design. *The Journal of Prosthetic Dentistry*.

[31] Shahmiri R., Aarts J. M., Bennani V., Das R., Swain M. V. (2013). Strain distribution in a Kennedy Class I implant assisted removable partial denture under various loading conditions. *International Journal of Dentistry*.

[32] Shahmiri R., Aarts J. M., Bennani V., Atieh M. A., Swain M. V. (2013). Finite element analysis of an implant-assisted removable partial denture. *Journal of Prosthodontics*.

[33] Verri F. R., Pellizzer E. P., Rocha E. P., Pereira J. A. (2007). Influence of length and diameter of implants associated with distal extension removable partial dentures. *Implant Dentistry*.

[34] Geckili O., Mumcu E., Bilhan H. (2012). The effect of maximum bite force, implant number, and attachment type on marginal bone loss around implants supporting mandibular overdentures: a retrospective study. *Clinical Implant Dentistry and Related Research*.

